# Heterologous Expression of a Novel Drug Transporter from the Malaria Parasite Alters Resistance to Quinoline Antimalarials

**DOI:** 10.1038/s41598-018-20816-0

**Published:** 2018-02-06

**Authors:** Sarah M. Tindall, Cindy Vallières, Dev H. Lakhani, Farida Islahudin, Kang-Nee Ting, Simon V. Avery

**Affiliations:** 1 School of Life Sciences, University of Nottingham, University Park, Nottingham, NG7 2RD UK; 2 Faculty of Pharmacy, Universiti Kebangsaan, Kuala Lumpur, 50300, Malaysia; 3 Department of Biomedical Sciences, University of Nottingham Malaysia Campus, Semenyih, Malaysia

## Abstract

Antimalarial drug resistance hampers effective malaria treatment. Critical SNPs in a particular, putative amino acid transporter were recently linked to chloroquine (CQ) resistance in malaria parasites. Here, we show that this conserved protein (PF3D7_0629500 in *Plasmodium falciparum*; AAT1 in *P*. *chabaudi*) is a structural homologue of the yeast amino acid transporter Tat2p, which is known to mediate quinine uptake and toxicity. Heterologous expression of PF3D7_0629500 in yeast produced CQ hypersensitivity, coincident with increased CQ uptake. PF3D7_0629500-expressing cultures were also sensitized to related antimalarials; amodiaquine, mefloquine and particularly quinine. Drug sensitivity was reversed by introducing a SNP linked to CQ resistance in the parasite. Like Tat2p, PF3D7_0629500**-**dependent quinine hypersensitivity was suppressible with tryptophan, consistent with a common transport mechanism. A four-fold increase in quinine uptake by PF3D7_0629500 expressing cells was abolished by the resistance SNP. The parasite protein localised primarily to the yeast plasma membrane. Its expression varied between cells and this heterogeneity was used to show that high-expressing cell subpopulations were the most drug sensitive. The results reveal that the PF3D7_0629500 protein can determine the level of sensitivity to several major quinine-related antimalarials through an amino acid-inhibitable drug transport function. The potential clinical relevance is discussed.

## Introduction

The fight for malaria eradication continues apace, but there were still over 200 million cases of this devastating parasitic disease in 2015^1,2^. In the absence of a commercially available vaccine, artemisinin combination therapies (ACTs) are the current main line of antimalarial defence in most countries. Quinoline antimalarials (commonly in combination with an antibiotic) are also recommended as first-line malaria treatments during early pregnancy and second line treatment for uncomplicated malaria cases, but remain first line drugs in many African countries^3–5^. Furthermore, quinoline derivatives such as amodiaquine, mefloquine and lumefantrine are currently used in recommended ACTs. Chloroquine was one of the most effective drugs ever produced and, along with primaquine, remains a drug of choice for treating *Plasmodium vivax* malaria^5^. Quinine (QN) has historically been a mainstay of the antimalarial drug repertoire but the wider use of QN is now hampered by poor compliance, the prevalence of adverse drug reactions and the availability of alternative antimalarials^3^.

One strategy in the battle against malaria is the identification of drug resistance mechanisms in the parasite. Identifying genetic changes that confer drug resistance helps the spread of resistance to be tracked and can allow appropriate antimalarial drug therapy to be tailored^6,7^. In addition, knowledge of the genetic basis for resistance can give insight to the mechanism of action of a drug, informing improved drug design or treatment strategies. Membrane transporters provide a classic example of proteins that can mediate drug resistance or sensitivity^8,9^. In the malaria parasite most lethal to humans, *Plasmodium falciparum*, multiple transporters have been associated with altered sensitivity to quinoline antimalarials including PfCRT, PfNHE1, PfMDR1 and PfMRP^10^. PfCRT is the most widely reported of these, localized to the parasite digestive vacuole and in which SNPs are commonly associated with chloroquine resistance^11^. Quinine resistance took over 200 years to emerge, but this is in striking contrast to other antimalarial drugs. Widespread resistance to chloroquine was evident just 40 years after its introduction. Quinine resistance is only found in some malaria-endemic areas and is usually low level^3^. The incidence of chloroquine resistance may sometimes be reversed relatively quickly when chloroquine treatment is discontinued^12,13^. Therefore, in the face of rising ACT resistance^14^ quinolines could in some regions continue to provide a valid alternative in the future.

One problem with characterisation of drug transport and resistance mechanisms in malaria parasites is that not all of the relevant species are easy to cultivate in the laboratory or to manipulate genetically, although improvements are being made including with *P*. *falciparum*^15,16^. Model organisms may be exploited as an alternative. The yeast *Saccharomyces cerevisiae* is an especially powerful model of eukaryotic cells that has been widely exploited for antimalarial drug discovery or mode-of-action studies^17–21^. Yeast has an unparalleled toolset for genetics and synthetic biology, and is a valuable host for heterologous expression of functional *Plasmodium* spp. proteins^22–24^. Previously, yeast genomic tools were used to reveal a novel mechanism of quinoline drug action, centred on cellular tryptophan (Trp) starvation. This action results from competition between drug and tryptophan for the high affinity yeast tryptophan/tyrosine transporter, Tat2p^20^. Subsequently, the link between tryptophan and quinine action was successfully extended to malaria patients, where it was found that individuals with higher plasma tryptophan levels had a low incidence of adverse reactions to quinine^25^. Furthermore, quinine perturbs biosynthesis and function of the major neurotransmitter serotonin, a metabolic product of tryptophan^19,26^.

In the present work, the earlier findings with yeast are exploited to test function of a Tat2p structural homologue that we identify in *Plasmodium* spp. It transpires that this homologue is a putative amino acid transporter in which SNPs were previously linked to chloroquine resistance in malaria parasites^27,28^. A recent attempt at characterisation by heterologous expression in *Xenopus laevis* oocytes did not produce detectably-functional protein^29^. Here we successfully apply a yeast heterologous expression system to show that the parasite protein mediates uptake of quinoline drugs so altering the level of drug resistance. The evidence suggests a new quinoline-drug transport protein, which may help explain the protein’s association with drug resistance of the parasite.

## Results

### The *P*. *falciparum* orthologue of *P*. *chabaudi aat1* and yeast *TAT2* mediates chloroquine uptake and toxicity

**Figure 1:**
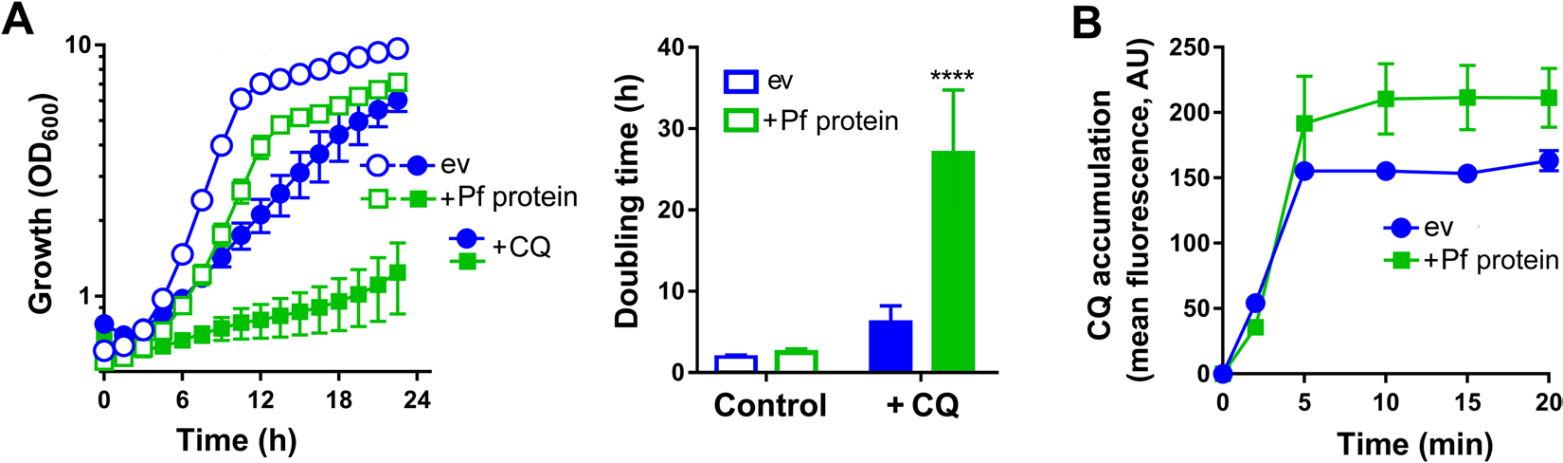
Expression of PF3D7_0629500 increases chloroquine uptake and toxicity. (**A**) Yeast *trp1Δ* cells transformed with pCM190 vector, either empty (ev) or containing the *PF3D7-0629500* ORF (Pf protein), were grown either with or without 1 mM chloroquine (CQ) (1 mM CQ was used in order to elicit yeast growth effects in the present assay system). Cell doubling times (right panel) were calculated from the exponential growth phase and the relevant treatments are as indicated on the panel. (**B**) Yeast *trp1Δ* cells transformed as in (**A**) were incubated in the presence of 0.4 mM chloroquine spiked with 20 µM LynxTag-CQ™. Cellular LynxTag-CQ™ was determined at intervals with flow cytometry, and cellular autofluorescence subtracted. AU, arbitrary units. All values are means ± SEM from three independent determinations. ****, *p* < 0.0001 according to multiple comparisons (with Tukey correction) by two way ANOVA.

The high affinity yeast tryptophan transporter Tat2p was previously found to transport quinine into cells, leading to quinine toxicity^20^. Here, standard BLAST searches for homologues of yeast Tat2p among *Plasmodium* spp. revealed no hits. However, an HHPRED homology search against Tat2p based on predicted secondary structures (see Methods) identified the putative amino acid transporter PF3D7_0629500 from *P*. *falciparum* (PlasmoDB: PFF1430c, Uniprot ID:C6KTD0, E-value 1.8e-17, Probability 99.87; note that E-value < 1 and probability > 95 indicate statistically significant homology: https://toolkit.tuebingen.mpg.de/hhpred/help_ov#evalues) (Supplementary Fig. S1). PF3D7_0629500 was 82^nd^ in a ranking of the proteins most-homologous to Tat2p among all available proteomes in HHPRED, and was the most significant homologue from *P*. *falciparum*. HHPred performs alignments of a protein amino acid sequence to secondary structure databases. No such database currently exists for certain species, such as the rodent parasite *P*. *chabaudi*, therefore we could not search Tat2p against all parasite species. However, PF3D7_0629500 is a known homologue of AAT1 from *P*. *chabaudi*, and a SNP in the *aat1* gene was previously linked with parasite resistance to chloroquine, a quinine derivative^27^. SNPs in *PF3D7_0629500* have also been associated with chloroquine resistance in *P*. *falciparum*^28^. Considering the evidence collectively, we hypothesized that the parasite protein may have a chloroquine and/or quinine transport function, resulting in toxicity if expressed heterologously in yeast. To test this, a codon optimised construct of the *PF3D7_0629500* ORF was cloned into the pCM190 expression vector. For heterologous expression of the parasite protein we capitalised on the availability of the yeast *trp1Δ* background. This strain is defective for tryptophan biosynthesis, similar to the parasite, and the strain’s dependency on exogenous tryptophan gives more sensitive detection of sensitivity to quinoline antimalarials^20^. Expression of PF3D7_0629500 in *trp1Δ* yeast conferred a chloroquine hypersensitivity phenotype (Fig. 1A). The cell doubling-time in the presence of CQ was ˃4-fold longer for cells expressing the parasite protein than empty vector control. In the absence of CQ, PF3D7_0629500 expression alone caused a small slowing of growth but the inhibitory effect attributable specifically to CQ remained considerably greater in these cells than in the empty vector control. To test whether the chloroquine sensitivity of PF3D7_0629500-expressing cells was related to increased chloroquine uptake, the chloroquine probe LynxTag-CQ™ was used to measure cellular chloroquine accumulation with flow cytometry. Chloroquine accumulation plateaued from ∼10 min. After 15 min, PF3D7_0629500-expressing cells had accumulated ∼38% more drug than empty-vector control cells (p < 0.05, Student’s t-test, one-tailed) (Fig. 1B). The results are consistent with the hypothesis that PF3D7_0629500 mediates elevated uptake of chloroquine, leading to drug hyper-sensitivity.

### Complementation of yeast Tat2 and tryptophan-sensitivity of PF3D7_0629500 activity

**Figure 2:**
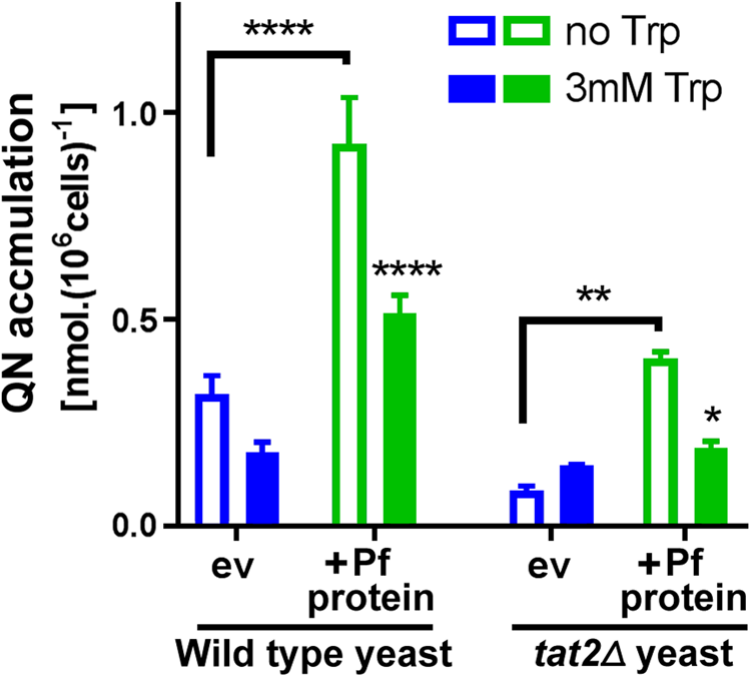
Complementation of yeast Tat2 and tryptophan-sensitivity of PF3D7_0629500 activity. Wild type or *tat2Δ* yeast cells transformed with pCM190 vector, either empty (ev) or containing the *PF3D7-0629500* ORF (Pf protein), were incubated for 20 min with 4 mM quinine either with or without 3 mM tryptophan (Trp). NaOH (6 mM) was included in all incubations to balance NaOH carry-over from tryptophan stock solution (NaOH had a small stimulatory effect on measured quinine uptake). Quinine analysis in cell lysates was according to absorbance determinations at 350 nm, normalised for cell numbers determined just before lysis, with subtraction of background (minus-quinine). *p < 0.05; **p < 0.01; ****p < 0.0001; according to multiple comparisons (Sidak’s test) by two way ANOVA.

The *trp1Δ* background used above, necessary to detect Tat2-suppressible quinoline sensitivity in yeast, was not suitable for testing complementation of Tat2 function by PF3D7_0629500 because a *trp1Δ/tat2Δ* deletant is inviable^20,30^. However, decreased uptake of quinine was previously demonstrated in the *tat2Δ* single-deletant^20,30^. Therefore, we used this phenotype to test complementation of Tat2 function by PF3D7_0629500. We used an assay based on quinine absorbance at 350 nm^31^, which produced a linear relationship over a range of quinine concentrations relevant to our assay (Supplementary Fig. S2A) and which demonstrated time-dependent saturation of uptake by cells (Supplementary Fig. S2B). The *tat2Δ* deletion mutant accumulated ∼75% less quinine than the wild type yeast (Fig. 2). This impairment of uptake was fully rescued by expression of the parasite protein (which also produced increased quinine uptake in the wild type yeast background). Previously, quinine sensitization mediated by the yeast tryptophan-permease Tat2 was shown to be suppressible by added tryptophan^20^. Here, inclusion of tryptophan (3 mM) significantly decreased PF3D7_0629500-dependent quinine uptake, measured in the *tat2Δ* background (Fig. 2). Therefore, PF3D7_0629500 could replace the tryptophan-suppressible quinine uptake activity of its yeast structural homologue.

### Sensitization to several quinoline antimalarials is suppressible with the T162E SNP or tryptophan

**Figure 3:**
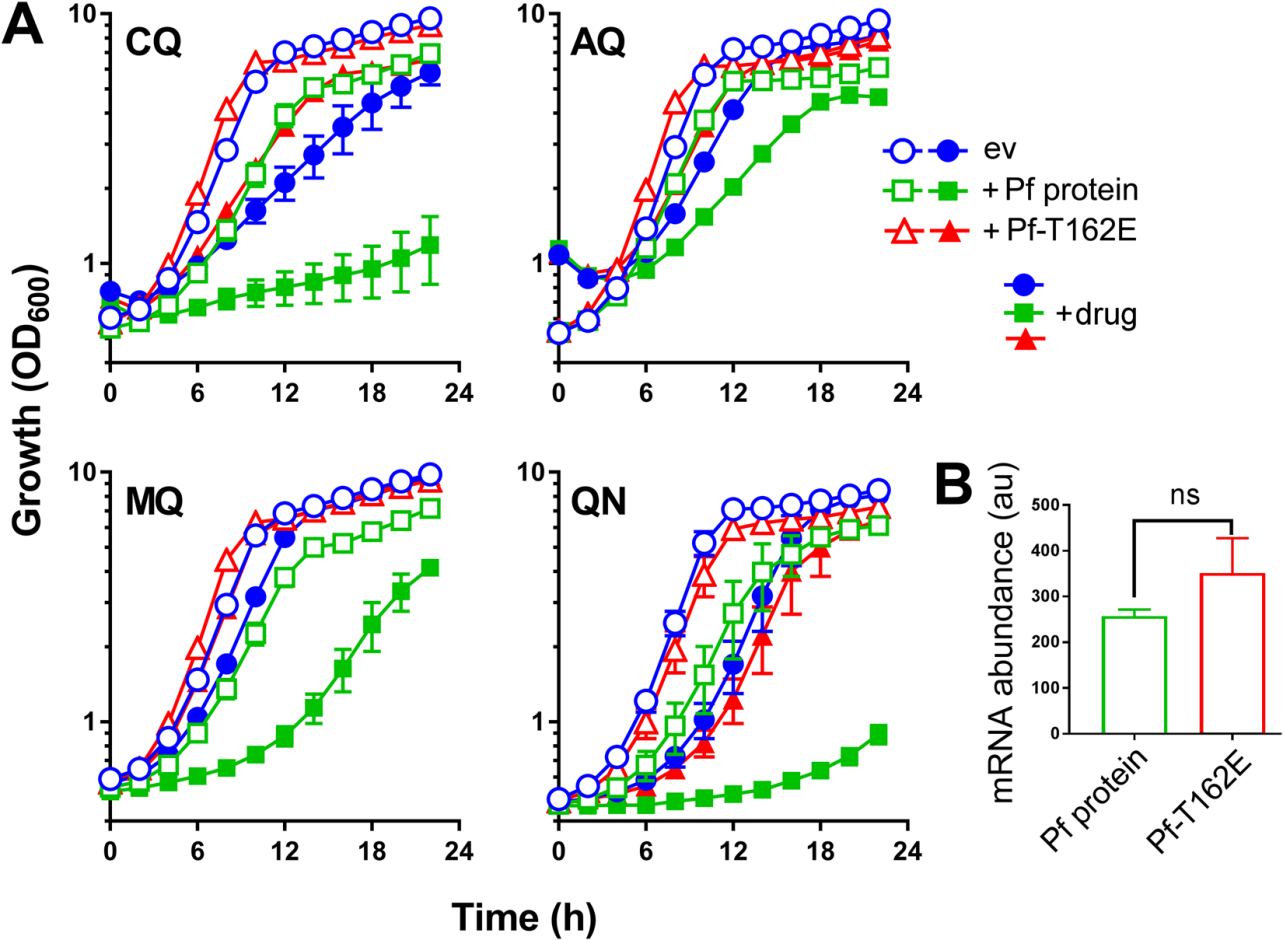
Sensitization to several quinoline antimalarials by PF3D7_0629500 expression and reversal with the T162E SNP. (**A**) Yeast *trp1Δ* cells transformed with pCM190 vector, either empty (ev) or expressing PF3D7-0629500 (Pf protein) or the same protein carrying the T162E SNP (Pf-T162E), were cultured with either 1 mM chloroquine (CQ), 1 mM amodiaquine (AQ), 25 μM mefloquine (MQ) or 3 mM quinine (QN). Mean data are shown from at least three independent experiments ± SEM. (**B**) RNA was extracted from wild type yeast transformed as in (**A**) and mRNA corresponding to the wild type *PF3D7-0629500* ORF or SNP (T162E) version was analysed with qRT-PCR, performed in triplicate for each condition. The same amount of RNA extract was used for each reaction. There was not a significant difference (ns, according to Student’s *t*-test, two tailed) between the conditions either when compared by raw counts (as shown) or after normalization against *ACT1* mRNA. au, arbitrary units.

We introduced a T162E mutation to PF3D7_0629500, which corresponded to the SNP previously linked to chloroquine resistance in *P*. *chabaudi*^27^. Furthering that association described in the parasite, yeast cells expressing the mutant construct were considerably more resistant to chloroquine than cells expressing the wild-type parasite protein. That is, introduction of the T162E SNP rescued CQ hypersensitivity of PF3D7_0629500-expressing cells (Fig. 3A). The SNP also rescued the mildly-slowed growth of the PF3D7_0629500-expressing cells seen in the absence of drug. The SNP version of the parasite gene was expressed at least as strongly in yeast as the wild type version. This was shown by qRT-PCR (Fig. 3B), and by measurement of expressed protein levels with western blotting or flow cytometry (Supplementary Fig. S3). (There was also no discernible difference in localization of the two versions of the protein; see below).

**Figure 4:**
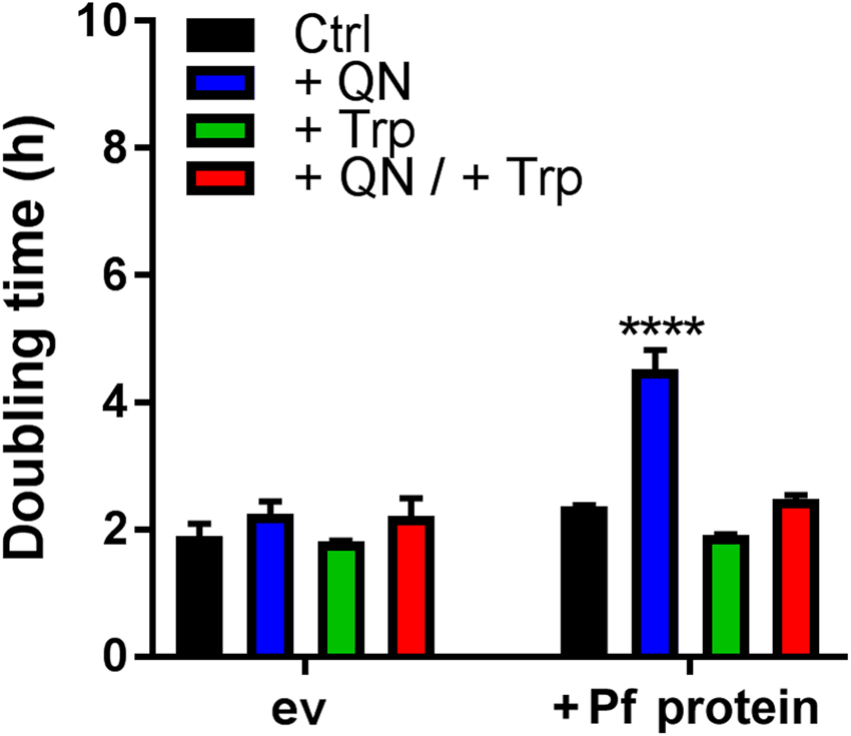
Sensitization to quinine in PF3D7_0629500-expressing cells is tryptophan suppressible. Yeast *trp1Δ* cells transformed with pCM190 vector, either empty (ev) or expressing PF3D7-0629500 (Pf protein), were cultured as specified either with or without 3 mM quinine and/or 1 mM tryptophan (Trp). Cell doubling times were calculated during the exponential phase of growth. Mean data are shown from at least three independent experiments ± SEM. ****p < 0.0001 according to multiple comparisons (with Tukey correction) by two way ANOVA.

We tested yeast expressing PF3D7_0629500 against several related drugs. As observed with chloroquine, PF3D7_0629500 expression conferred hypersensitivity to amodiaquine (weakest phenotype), mefloquine and quinine (strongest phenotype). This indicates that the parasite protein determines sensitivity to multiple quinolines. In all cases, the SNP corresponding to that identified previously in the parasite reversed the sensitivity phenotypes (Fig. 3A). IC_50_ values were derived from growth rate data at varying doses of the drugs which gave the strongest phenotypes above. For cells expressing empty vector, PF3D7_0629500 or the SNP construct, respectively, IC_50_ values were 3.56, 2.92 or 3.42 mM for quinine and 0.694, 0.435 or 1.037 mM for chloroquine. (In the case of chloroquine, cells expressing the T162E SNP construct were even more resistant to drug than cells expressing empty vector.) Previously, as with quinine uptake (above), quinine sensitization mediated by the yeast Tat2 was shown to be suppressible by tryptophan^20^. Here, inclusion of tryptophan in the medium also suppressed PF3D7_0629500-mediated sensitization to quinine (Fig. 4), further supporting a similar competition between drug and amino acid at this proposed transporter from the parasite.

### Relationship between quinine uptake, quinine sensitivity and membrane-localization of PF3D7_0629500 in individual cells

**Figure 5:**
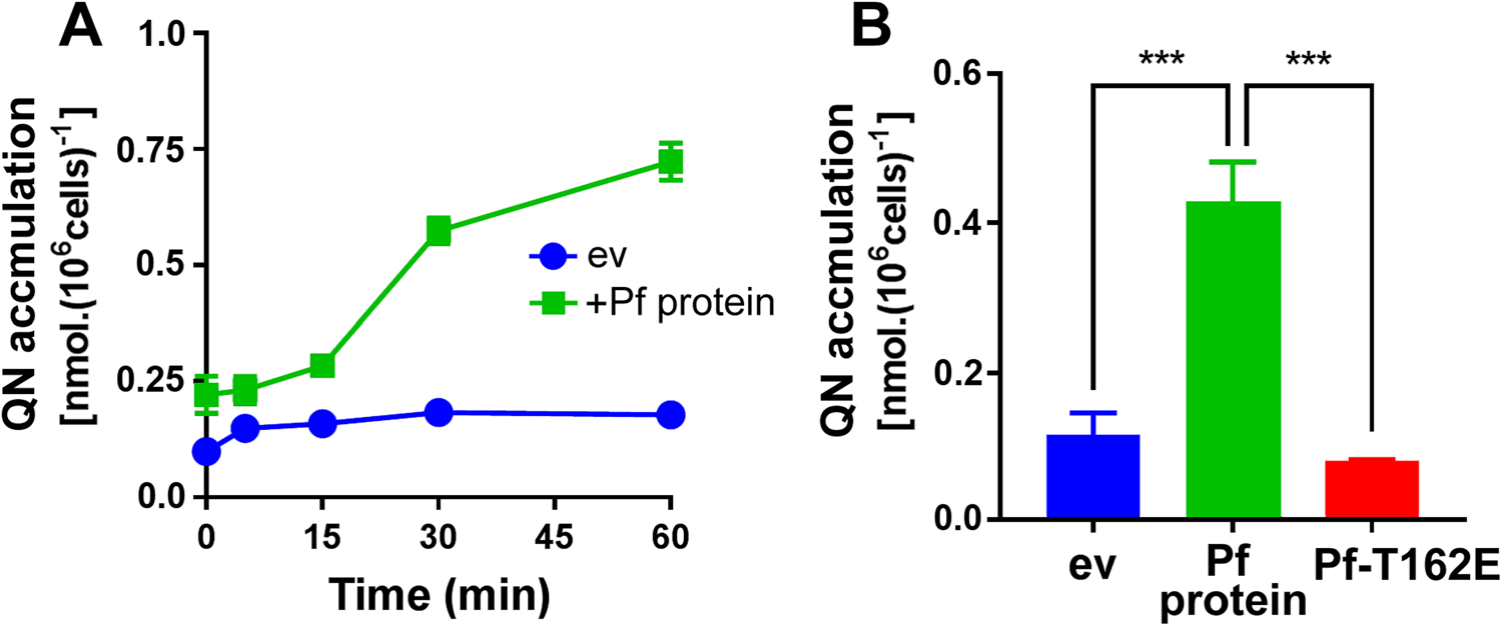
Increased quinine uptake in drug-sensitive PF3D7_0629500-expressing cells and reversal with the T162E SNP. (**A**) Yeast *trp1Δ* cells transformed with vector pCM190, either empty (ev) or expressing PF3D7-0629500 (Pf protein), were cultured with 4 mM quinine for the indicated time intervals before harvesting and lysis. Quinine analysis was as described in the Fig. 2 legend. (**B**) Yeast *trp1Δ* cells transformed with vector pCM190, either empty (ev), or expressing PF3D7-0629500 (Pf protein) or the same protein carrying the T162E SNP (Pf-T162E), were cultured for 20 min with 4 mM quinine before analysis of cellular quinine. Values are means ± SEM from three independent determinations. ***, *p* < 0.001 according to multiple comparisons (with Tukey correction) by two way ANOVA.

Quinine uptake was assayed in the *trp1Δ* yeast background (versus *tat2Δ* background in Fig. 2 above) so we could test whether quinine sensitization (Fig. 3A) was correlated with increased drug uptake in PF3D7_0629500-expressing cells, as was the case with chloroquine (Fig. 1). A rapid (≤30 s) initial association of quinine with cells (probably reflecting non-specific cell surface binding) was followed by continued accumulation of the drug in the PF3D7_0629500-expressing culture, but not in the empty vector control (Fig. 5A). After 60 min, cells expressing the parasite protein had accumulated ∼4-fold more drug. This increased uptake was abrogated by introduction of the T162E SNP (Fig. 5B). The data were consistent with the suggestion that increased drug uptake in PF3D7_0629500-expressing cells causes their quinine sensitivity.

**Figure 6:**
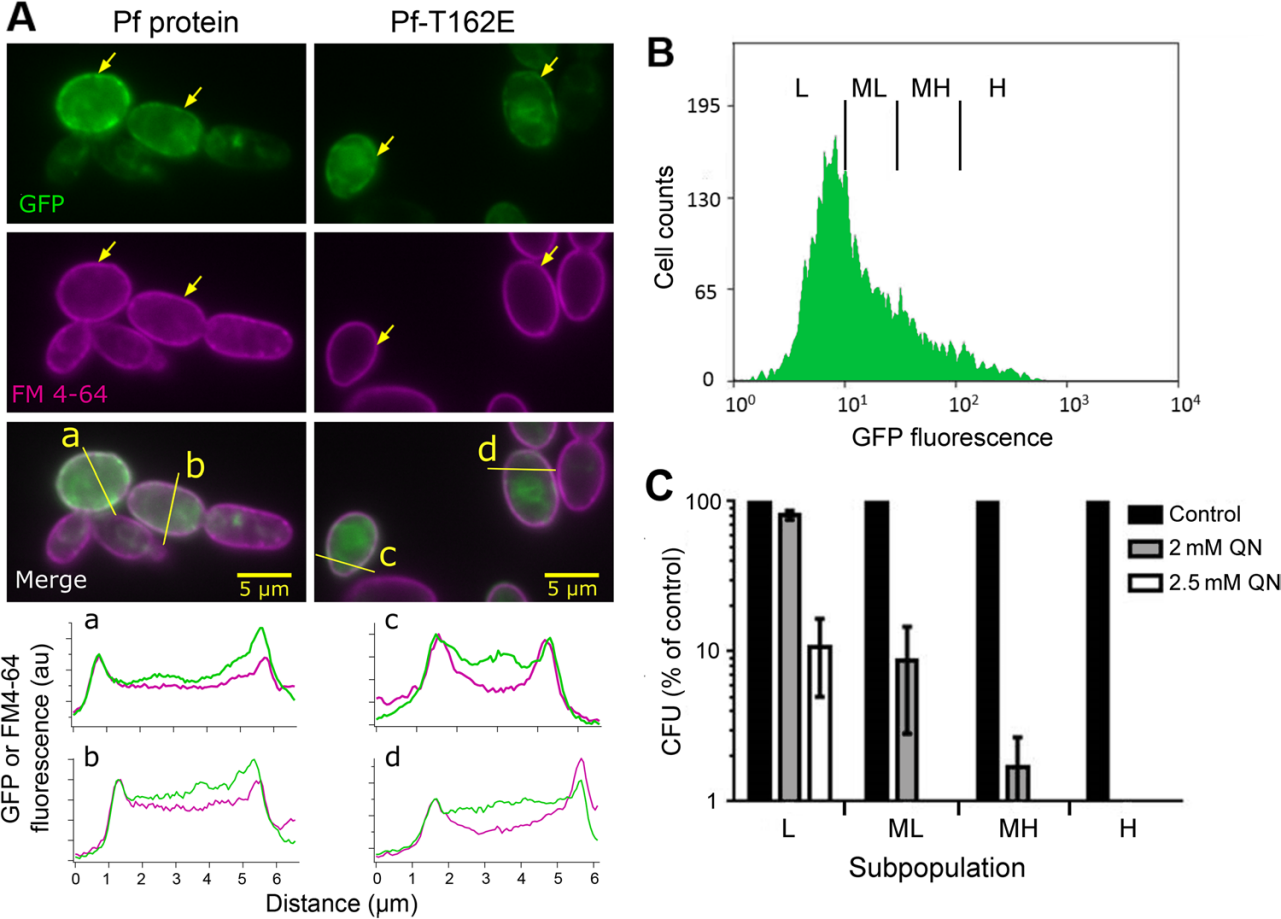
Heterogeneous expression of PF3D7_0629500 at the yeast plasma membrane determines individual-cell drug sensitivity. (**A**) Yeast *tat2Δ* cells expressing GFP-tagged PF3D7-0629500 (Pf protein) or Pf-T162E from vector pCM190 were stained with FM4-64 and examined by fluorescence microscopy. The fields of view shown are representative of several observed. Merged images (GFP in green, FM4-64 marker in magenta) are shown, as are the individual channels. Intensity line profiles along the lines (a, b, c, d) shown in the merged images are presented in the lower panels. Arrows indicate the high-expressing cells. au, arbitrary units. (**B**) Yeast *trp1Δ* cells expressing GFP-tagged PF3D7-0629500 were sorted into low (L), medium-low (ML), medium-high (MH) and high (H) -expressing cell subpopulations, according to GFP fluorescence; > 1,000 cells were sorted from each subpopulation. (**C**) Sorted cell subpopulations (**B**) were spread plated to YPD agar supplemented or not with quinine as indicated. Colony forming units (CFUs) were enumerated after 4 d incubation and expressed as a percentage of CFUs observed for the same cell subpopulation on minus-quinine control plates. Values are means ± SEM from three independent experiments.

Yeast Tat2p is a plasma membrane-localized transport protein^32^. In *P*. *falciparum*, PF3D7_0629500 has been reported to localize to the digestive vacuole membrane, observed using a fluorescent reporter fusion construct, in keeping with its putative function as an amino acid transporter^33^. To localize PF3D7_0629500 in yeast we expressed a GFP tagged version of the protein in the *tat2Δ* yeast background. Consistent with a transport function, the protein localized primarily to the yeast plasma membrane, co-localizing precisely with the membrane stain FM4-64 (a short FM4-64 staining time was used to restrict staining to the plasma membrane^34^) (Fig. 6A). There was no apparent difference in localization of the PF3D7-0629500 protein versus the same protein carrying the T162E SNP. The PfCRT transporter of the parasite’s digestive-vacuole also localizes to the plasma membrane when expressed in yeast^24^. Unexpectedly, there was visible heterogeneity between individual cells in their expression-level and localization of the protein: high-expressing cells are indicated by arrows, while comparison with the FM4-64 panels highlights certain cells that showed little or no PF3D7_0629500-associated GFP fluorescence (Fig. 6A). Heterogeneity of gene expression, protein localization or other phenotype between individual cells within genetically-uniform populations is described in other systems^35–37^. Here, we exploited this phenomenon to interrogate further the relationship between PF3D7_0629500 expression-level and drug sensitivity. Again we exploited the *trp1Δ* background to help discern drug sensitivity. Heterogeneity in expression of PF3D7_0629500-GFP was quantifiable with flow cytometry, which indicated a broad non-normal distribution of cell fluorescences extending to almost two orders of magnitude greater than the mode cell-fluorescence (Fig. 6B). The > 100-fold total variation in expression level was comparable to that of one of the most variably expressed yeast proteins (a virulence factor of *Candida glabrata)* reported to date^38^. Cell subpopulations were gated by their level of GFP fluorescence (Fig. 6B), then FACS sorted into four cell suspensions which were each assayed independently for quinine resistance. Quinine resistance was progressively eroded with increasing expression of PF3D7_0629500-GFP (Fig. 6C). The highest expressing fraction of cells showed no growth at 2 mM quinine. In contrast, the lowest expressing cells retained > 80% outgrowth at 2 mM quinine, and ∼10% outgrowth at 2.5 mM quinine. Results for medium expression-level populations supported this trend, albeit a little more variable between replicates. Therefore, reinforcing the observations with bulk cell populations (Fig. 3A), variation in single-cell expression level of PF3D7_0629500 affects the drug sensitivities of individual cells. Such variation itself can have important implications, as discussed further below.

## Discussion

Previously it was found that, in yeast, quinine is specifically transported via the high-affinity aromatic amino acid transporter Tat2^20^. The present study extrapolated this work to the *Plasmodium* parasite. The major finding from heterologous expression studies with PF3D7_0629500, a Tat2 structural-homologue identified from *P*. *falciparum*, was that the parasite protein determines quinine and chloroquine uptake and resistance when expressed in yeast. This homologue could not be identified using a standard BLAST search but was found with the HHPred tool which uses predicted secondary structures. Protein secondary structures diverge at a slower rate than amino acid sequence and so can help to identify more distant homologues^39,40^.

The protein that is the focus of this work has previously been associated with quinoline resistance in the parasite. PF3D7_0629500 was identified by transcriptome profiling as one of only 45 genes differentially expressed between chloroquine-sensitive and -resistant mutants of *P*. *falciparum*^41^. Moreover, a non-synonymous SNP in AAT1 (the PF3D7_0629500 homologue of *P*. *chabaudi*) was a key determinant of chloroquine resistance in laboratory evolved parasites^27^, and PF3D7_0629500 was recently associated with chloroquine resistance in *P*. *falciparum* by genome-wide association^28^. However, the protein has not been subject to detailed study and its function is not previously established. Function could not be detected following heterologous expression in *Xenopus laevis* oocytes^29^, in contrast to the yeast expression system described here. Reasons for this difference may include differences in protein folding or localization in the two expression systems. Reported vacuolar-membrane localization of the protein in the parasite translated into plasma membrane localization in yeast (see below), which was effective as the protein was functional. Yeast is well documented as a suitable host for heterologous expression of functional *Plasmodium* spp. proteins^22–24^. The PF3D7_0629500 protein is expressed throughout the parasite intraerythrocytic cycle, at which most current antimalarials act^42,43^, and is designated a putative amino acid transmembrane transporter based on sequence similarity. The protein has been reported to be expressed at the parasite’s digestive vacuole membrane^33^. Heterologous expression of the GFP tagged version in yeast gave localization primarily to the plasma membrane, providing a convenient system for assaying transport function via analysis of whole-cell drug contents after simple cell separation from medium.

**Figure 7:**
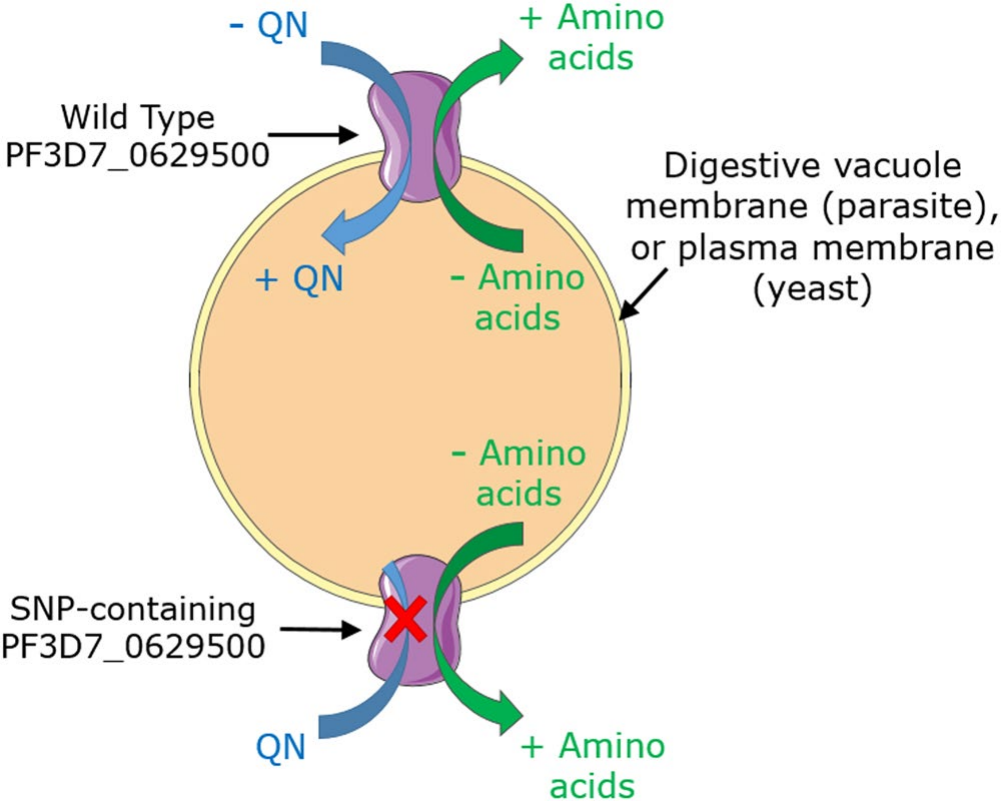
A model of PF3D7_0629500 action. PF3D7_0629500 is proposed to facilitate cross-membrane diffusion of amino acids or structurally-related quinolines down concentration gradients. In the parasite, this would probably enable release of amino acids from the digestive vacuole or entry of drug into the vacuole. In yeast, where the heterologous protein localizes to the plasma membrane, PF3D7_0629500 enables drug uptake into cells. The T162E SNP abrogates the drug transport function, decreasing drug accumulation at the respective sites of action in both organisms.

The localization information helps rationalise the effects of the protein on drug resistance. In the parasite, PF3D7_0629500 is likely to mediate transport of a wide range of amino acids or small peptides from the parasite’s digestive vacuole, where haemoglobin is digested^42,44^. Such movement down the concentration gradient from vacuole to cytoplasm is consistent with a facilitated diffusion transport mechanism, as occurs in the yeast homologue Tat2. This is further supported by suggestions that PF3D7_0629500-mediated drug transport is passive, unaffected by incubation at 4 C or treatment with the protonophore CCCP (S. Tindall and S.V. Avery, unpublished data). It follows that, according to its localization, PF3D7_0629500 would facilitate transport of drug (down the concentration gradient) either from cytoplasm to vacuole in the parasite, or from extra- to intra-cellular in yeast (Fig. 7). In both cases, this represents transport of drug to its anticipated site of action (different in yeast and parasite) and is in keeping with the drug-sensitivity or -resistance phenotypes seen, respectively, with expression of the wild type or SNP (loss of drug transport) versions of the protein in yeast (present data) and parasite^27^. The SNP introduced here corresponded to that found in the parasite-resistance study and which, we showed, impairs drug-transport function. The T162E SNP creates a more negative charge in a conserved region near the start of a transmembrane helix; a very similar effect to that of the K76T SNP in PfCRT which confers CQ resistance^27^, discussed further below.

As with the yeast Tat2p transporter^20^, PF3D7_0629500-dependent quinine sensitivity was suppressible with tryptophan. This suggests that as in yeast, the drug competes for uptake with tryptophan, a proposed natural substrate of the parasite protein. Such competition may be less relevant where drug and amino acid are moving down concentration gradients in opposite directions. Nonetheless, where it does occur, competition can be ascribed to the structural similarity of tryptophan and quinine, a drug that is derived enzymatically from tryptophan^45^. Competition between quinine and tryptophan also raises the possibility that quinine displaces the essential amino acid intracellularly, e.g. during metabolism or protein synthesis^20^. Tryptophan depletion arising in this way has been proposed to account for certain of the drug’s adverse effects in quinine-treated malaria patients^25^. It cannot be discounted that a similar tryptophan-depletion mechanism could contribute to quinine action in the parasite.

There was heterogeneity between cells in the level of GFP tagged PF3D7_0629500 expression in yeast. Such heterogeneity underscores how population averaged measurements can misrepresent the activities relevant to any individual cell^46^. Phenotypic heterogeneity within genetically-uniform cell populations is thought to be a universal phenomenon, which has received increased scrutiny in recent years with the growing awareness of its potential role in the persistence of microbial infections and tumours^38,47,48^. Typically, phenotypic heterogeneity within a clonal cell population is caused by gene-expression variation, arising from noise during transcription or translation, or cell cycle-, age-, or epigenetically-driven changes in expression. Epigenetic changes in the expression of surface antigens of *P*. *falciparum* are reported to help avoid host immune responses^35^. The marked heterogeneity of PF3D7_0629500 expression seen in this study was exploited as a novel tool to dissect the relationship between drug sensitivity and PF3D7_0629500 expression, at an individual cell level. We cannot infer whether PF3D7_0629500 expression or membrane-localization is as heterogeneous in the parasite as is apparent in yeast. However, given the protein’s evident function in quinoline-drug transport and toxicity, any heterogeneity could have important implications for malaria treatment with quinolines. In bacteria, phenotypic heterogeneity is well known to create phenotypically resistant sub-populations – persister cells – which may re-initiate infection when antimicrobial therapy is stopped^48^. To date there has been less work of a similar nature in *Plasmodium* spp., although “dormancy” in the parasite could have a similar effect as antimicrobial persistence^49^. The present results suggest a possibility that PF3D7_0629500 might be a good candidate for further study. Moreover, gene expression heterogeneity within clonal *Plasmodium* spp. populations could be an important gap in current drug resistance models.

Several parallels have previously been noted between PF3D7_0629500 and PfCRT, the best studied chloroquine resistance determinant in *P*. *falciparum*. Both are thought to serve as channel proteins on the digestive vacuole membrane, each containing 10 transmembrane domains^27,50^. Both may be involved in the transport of amino acids or small peptides^42,51^. Furthermore, inhibition of PfCRT-mediated amino acid and peptide transport by chloroquine has been suggested potentially to contribute to the drug’s inhibitory action^51^. A similar potential action is discussed above for PF3D7_0629500. Finally, mutations in PfCRT have been shown to alter sensitivity to further quinolines, such as quinine, amodiaquine and mefloquine^52,53^. PF3D7_0629500 expression sensitized yeast to all the quinoline antimalarials that were tested in this study. The evidence suggests that PF3D7_0629500 could be important as a multi-drug sensitivity/resistance determinant in *Plasmodium* spp.

The weight of published evidence remains with PfCRT (in particular the K76T SNP) as the foremost marker of chloroquine resistance in isolates of *P*. *falciparum*. A similar strong marker has not been found with the *P*. *vivax* homologue (PvCRT)^54,55^, although there is evidence that chloroquine resistance may be conferred by changes in levels of PvCRT (or PvMDR1) expression^56^. It would be of interest to investigate the *P*. *vivax* orthologue of *PF3D7_0629500* (*PVP01_1120000*) as a potential resistance marker in *P*. *vivax*, where resistance to chloroquine is a growing concern^57^.

Among the current malaria treatment options, quinolines are commonly combined with artemisinin (or artemisinin derivative) in antimalarial combination treatments (ACTs). Therefore, it is worth noting that a SNP in PF3D7_0629500 (S258L) has previously been associated with artemisinin-resistant subpopulations of clinical *P*. *falciparum* isolates^7^. Any evolutionary selection of this SNP is not necessarily artemisinin-driven, as mutations conferring artemisinin resistance can be selected before a population has been exposed to the drug^58^. Moreover, given the present data and considering the prevalence of ACT therapy, we also suggest the possibility that selection for the S258L SNP could have been driven by quinolines used in combination with artemisinin.

In conclusion, rationalising previous observations with malaria parasites, the heterologous expression studies presented here reveal that PF3D7_0629500 activity can determine the transport and action of multiple quinoline drugs. Furthermore, cell-cell heterogeneity in PF3D7_0629500 activity provided a novel tool to corroborate that relationship, while suggesting the tantalising possibility of heterogeneous activity also in the parasite and attendant implications for modelling quinoline drug resistance. Finally, the results reinforce the value of model systems for uncovering or substantiating novel protein functions that may have an important bearing on the spread (and control) of antimalarial drug resistance.

## Methods

### Bioinformatic analysis

The online tool HHPRED^40^ (available at http://toolkit.tuebingen.mpg.de/hhpred) was used to find orthologues of the *S*. *cerevisiae* high-affinity tryptophan transporter, Tat2, in *P*. *falciparum*. The Tat2 amino acid sequence from *S*. *cerevisiae* (UniProt P38967) was used as a query sequence in HHPRED using the *Plasmodium falciparum* and *Saccharomyces cerevisiae* databases as the target proteomes. All other options were at default settings. This seed query generated a multiple alignment of homologues using multiple iterations of PSI-BLAST. A secondary structure prediction was carried out and annotated on the final alignment using PSIPRED^59^ from which a profile Hidden Markov Model (HMM) is derived. HMM-to-HMM comparisons were carried out against all available HMM databases in the target proteomes to locate homologues based on similarity of predicted secondary structure rather than sequence alone.

### Yeast strains and culture conditions

The *S*. *cerevisiae* diploid strain BY4743 (*MAT*a/*MAT*α *his3-*1/*his3-*1 *leu2-*0*/leu2-*0 *met15-*0/*MET15 LYS2*/*lys2-*0 *ura3-*0/*ura3-*0), and isogenic deletion mutants *trp1Δ* and *tat2Δ* were from Euroscarf (Frankfurt, Germany). Yeast were maintained and grown in YPD medium (2% peptone, 1% yeast extract, 2% D-glucose; Oxoid, Basingstoke, UK), or YNB medium (0.69% yeast nitrogen base without amino acids; Formedium; Norfolk, UK) supplemented with 2% (*w/v*) D-glucose and as appropriate for plasmid selection^60^. Where necessary, media were solidified with 2% (*w/v*) agar (Sigma). To culture organisms for experiments, single colonies were used to inoculate broth cultures in Erlenmeyer flasks and incubated at 30 °C with orbital shaking at 120 rev min^−1^. The same procedure was used for all strains.

### Growth inhibition assays

For continuous growth assays with the different yeast strains, mid/late-exponential cultures were diluted to OD_600_∼0.1 and 300 µl aliquots transferred to 48-well microtiter plates (Greiner Bio-One; Stonehouse, UK) with antimalarial drugs added as specified and balanced for any solvent additions. Plates were incubated at 30 °C with shaking in a BioTek Powerwave XS microplate spectrophotometer and OD_600_ was recorded every 30 min. Cell doubling times were calculated from the linear portion of exponential growth. Drug concentrations giving 50% growth inhibition (IC_50_ values) were determined from doubling-time data at different drug concentrations. Antimalarial drugs used were amodiaquine dihydrochloride dihydrate (AMQ), chloroquine diphosphate salt (CQ), mefloquine hydrochloride (MQ) and quinine dihydrochloride (QN) (Sigma). Drugs were dissolved in water except quinine and mefloquine which were prepared in 70% (v/v) ethanol stock solutions, diluted to 0.5% final ethanol concentration for experiments. Ethanol at 0.5% has no effect on yeast viability and was balanced in relevant control incubations. Tryptophan additions were from stock solutions of 0.5 M L-tryptophan (Sigma) prepared in 1 M NaOH. NaOH (6 mM) was included in relevant control incubations to balance NaOH carry-over from the tryptophan stock solution. For assays based on colony forming ability, FACS-sorted cell subpopulations (see below) were diluted in PBS to OD_600_∼5 × 10^−5^ (∼1500 cells ml^−1^) and 200 µl aliquots spread-plated to YPD agar plates supplemented with QN as specified. Colony forming units (CFUs) were counted after 4 d incubation at 30 °C.

### Heterologous expression of PF3D7_0629500 and Introduction of SNPs

A yeast-codon optimised *PF3D7_0629500* gene cloned in the pUC57 vector was a kind gift from Enrique Salcedo-Sora (Liverpool Hope University). For expression in yeast, *Not*I and *Pme*I sites were added to the 5′ and 3′ termini of the *PF3D7_0629500* ORF by PCR and the product ligated between these restriction sites in the pCM190 vector^61^. This placed the ORF under the control of the doxycycline-regulatable *tetO* promoter. To introduce a T162E SNP into PF3D7_0629500, the Q5 site-directed mutagenesis kit was used according to the manufacturer’s instructions [New England Biolabs (NEB); Hitchin, UK]. Introduction of the SNP was verified by sequencing. Recombinant plasmids were transformed into *S*. *cerevisiae* using the lithium acetate method^62^. To tag PF3D7_0629500 and PF3D7_0629500^T162E^ with GFP, *Bst*BI and *Asc*I sites were added to the 5′ and 3′ termini of the relevant ORF by PCR and the product ligated between these restriction sites in the pFA6a-GFP(S65T)-His3MX6 vector^63^. The EGFP cassette with a fragment of the *PF3D7_0629500* or *PF3D7_0629500*^*T162E*^ ORF up to its 3′ *Blp*I digestion site was then PCR amplified with the addition of a 5′ *Sfi*I site and ligated in frame to pCM190-PF3D7_0629500. Primer sequences are available on request. Transformed yeast were grown on YNB medium with appropriate supplements for selection. All DNA cloning and genetic manipulations were performed in *Escherichia coli* XL1-blue cells. PCR, restriction digests and ligations were carried out using standard protocols^64^.

### RNA extraction and quantitative RT-PCR (qRT-PCR)

mRNA from specified genes in plasmid-transformed *S*. *cerevisiae* BY4743 was quantified by qRT-PCR exactly as described previously^65^, except that RNA was isolated by the “hot phenol” technique then treated with Amplification Grade DNase I (Sigma-Aldrich, St. Louis, MO), and 25 ng cDNA with 175 nM gene*-*specific primers (sequences available on request) were used in the PCR reactions. PCRs were carried out for 40 cycles; denaturation at 95 °C for 15 s, annealing/extension at 60 °C for 30 s. Melting-curve analysis confirmed a single PCR product. Amplification was quantified from a standard curve constructed from reactions with defined genomic DNA concentrations.

### Fluorescence microscopy and FACS

For examination of GFP fluorescence by microscopy and flow cytometry, exponential-phase yeast cells were washed with PBS, and imaged with a DeltaVision Elite microscope (GE Healthcare Life Sciences, UK) equipped with a Photometrics CoolSnap HQ2 camera (Photometrics, USA), or analysed with a Beckman Coulter FC500 cytometer. Staining for ∼5 min with FM4-64 (SynaptoRed reagent; Calbiochem, EMD Biosciences, San Diego, CA) was performed as described previously^34^. Microscopic images were acquired with a 100 × 1.4 NA objective lens. GFP fluorescence was captured using the FITC filter set, and FM4-64 using the TRITC filter for excitation and the Cy-5 filter for emission; the Quad polychroic was used for both channels. Exposure times were the same for the different strains; 0.4 s and 0.05 s for the FITC and Cy-5 channels, respectively. Images were collected in a single z-plane. Images, line profiles and landmarks were produced in Fiji (https://imagej.net/Fiji) and Igor Pro (Wavemetrics, USA) and images assembled in Inkscape (http://www.inkscape.org/). To FACS-sort cells, yeast expressing PF3D7_0629500-GFP were harvested by centrifugation (3,220 *g*, 3 min) and resuspended in PBS at OD_600_∼2.0, before gating and sorting with a Beckman Coulter MoFlo XDP flow cytometer, equipped with a 488 nm laser. Emitted GFP fluorescence was collected using a 529/28 nm band pass filter. FACS-sorted cell subpopulations were diluted in PBS and spread to YPD agar as described above.

### Preparation of protein extracts and western blotting

Cells were collected by centrifugation, washed serially with cold water and lysis buffer (50 mM Tri-HCl, 500 mM NaCl, pH 7.4, supplemented with protease inhibitors: 1 mM PMSF, 4 mM benzamidine hydrochloride, 2.5 mM EDTA, pH 8) then disrupted with glass beads^66^. Lysates were treated with 1% Triton X-100 on ice for 30 min and then with cracking buffer (8 M Urea, 5% (w/v) SDS, 40 mM Tris-HCl pH 6.8, 0.1 mM EDTA, 0.4 mg/ml bromophenol blue) at 37 °C for 10 min followed by incubation at 95 °C for a further 10 min. For western blotting, proteins were separated by electrophoresis on 10% (w/v) NuPAGE Bis-Tris gels (Life Technologies) before transfer to nitrocellulose membrane (GE Healthcare). Protein loading was shown by staining with Ponceau S (Sigma). Immunodetection of GFP tagged proteins was with anti-GFP primary antibody (1:1000 dilution; Roche) and poly horseradish peroxidase (poly HRP) conjugated goat anti-mouse antibody (1:10000 dilution; Thermo Scientific). GFP tagged proteins were detected with an electrochemiluminescence HRP kit (Pierce) and imaged using a Chemidoc XRS (Bio-Rad). Protein band intensities were quantified with ImageJ software.

### Assays of drug uptake

Quinine uptake was assayed essentially as described previously^67^, except that quinine absorbance at 350 nm^31^ was measured instead of quinine fluorescence. Briefly, overnight cultures were diluted to OD_600_ 0.1 in fresh YPD medium and cultured for a further 4 h with shaking. Quinine was added to a final concentration of 4 mM and cells incubated at 30 °C, 120 rev min^−1^. At intervals, cells were harvested by centrifugation (3,220 *g*, 3 min), washed three times with ice cold water and resuspended in 10% (*w/v*) perchloric acid, 2 M sodium methanesulfonate together with an equal volume of acid-washed glass beads (425–600 µm, Sigma). Cells (∼3.7 × 10^8^ in 800 μl) were lysed by 3 × 1-min vortexing with beads interspersed with 1 min incubations on ice, centrifuged at 16,060 *g*, 5 min, before 20 μl supernatant (corresponding to lysate from ∼1 × 10^7^ cells) was diluted with 180 μl lysis buffer and *A*_350_ measured with an Ultrospec 2000 UV/visible spectrophotometer (Amersham Pharmacia Biotech; Amersham, UK). Values for *A*_350_ were normalised against OD_600_ determinations taken just before cell lysis. (The OD_600_ determinations provided estimates of the cell concentrations.) Chloroquine uptake by cells was estimated using a fluorescently-labelled chloroquine molecule, LynxTag-CQ™ Green (BioLynx Technologies), as described previously^68^. Fluorescence from cellular LynxTag-CQ™ Green was measured with a Beckman Coulter FC500 flow cytometer, with excitation at 488 nm. Cell autofluorescence was subtracted.

### Data availability

No large datasets were generated or analysed during the current study. Other data are available from the author on reasonable request.

## Electronic supplementary material

Supplementary material

## References

[1] Cowman AF, Healer J, Marapana D, Marsh K (2016). Malaria: Biology and disease. Cell.

[2] WHO. Fact Sheet: World Malaria Report 2015. *Retrieved 23 November 2016* (2015).

[3] Achan J (2011). Quinine, an old anti-malarial drug in a modern world: role in the treatment of malaria. Malaria J..

[4] Hill J (2014). Women’s access and provider practices for the case management of malaria during pregnancy: a systematic review and meta-analysis. PLos Med..

[5] WHO. *Guidelines for the Treatment of Malaria - third edition* (2015).

[6] Henriques G (2015). The mu-subunit of *Plasmodium falciparum c*lathrin-associated adaptor protein 2 modulates *in vitro* parasite response to artemisinin and quinine. Antimicr. Ag. Chemother..

[7] Miotto O (2013). Multiple populations of artemisinin-resistant *Plasmodium falciparum* in Cambodia. Nat. Genet..

[8] Chang G (2003). Multidrug resistance ABC transporters. FEBS Lett..

[9] Kell DB, Oliver SG (2014). How drugs get into cells: tested and testable predictions to help discriminate between transporter-mediated uptake and lipoidal bilayer diffusion. Front. Pharmacol..

[10] Petersen I, Eastman R, Lanzer M (2011). Drug-resistant malaria: Molecular mechanisms and implications for public health. FEBS Lett..

[11] Ecker A, Lehane AM, Clain J, Fidock DA (2012). PfCRT and its role in antimalarial drug resistance. Trends Parasitol..

[12] Huang B (2016). Prevalence of *crt* and *mdr-1* mutations in *Plasmodium falciparum* isolates from Grande Comore island after withdrawal of chloroquine. Malaria J..

[13] Mwai L (2009). Chloroquine resistance before and after its withdrawal in Kenya. Malaria J..

[14] Fairhurst RM, Dondorp AM (2016). Artemisinin-resistant *Plasmodium falciparu*m malaria. Microbiol. Spectr..

[15] Ganesan SM, Falla A, Goldfless SJ, Nasamu AS, Niles JC (2016). Synthetic RNA-protein modules integrated with native translation mechanisms to control gene expression in malaria parasites. Nature Comm..

[16] Ghorbal M (2014). Genome editing in the human malaria parasite *Plasmodium falciparum* using the CRISPR-Cas9 system. Nature Biotech..

[17] Balana-Fouce R, Reguera RM (2016). Yeast-based systems for tropical disease drug discovery. Expert Op. Drug Discov..

[18] dos Santos SC, Sa-Correia I (2011). A genome-wide screen identifies yeast genes required for protection against or enhanced cytotoxicity of the antimalarial drug quinine. Mol. Genet. Genom..

[19] Islahudin F (2014). The antimalarial drug quinine interferes with serotonin biosynthesis and action. Sci. Rep..

[20] Khozoie C, Pleass RJ, Avery SV (2009). The antimalarial drug quinine disrupts Tat2p-mediated tryptophan transport and causes tryptophan starvation. J. Biol. Chem..

[21] Li W (2005). Yeast model uncovers dual roles of mitochondria in the action of arternisinin. PLoS Genet..

[22] Bilsland E (2011). Functional expression of parasite drug targets and their human orthologs in yeast. PLoS Negl. Trop. Dis..

[23] Slavic K (2016). A vacuolar iron-transporter homologue acts as a detoxifier in *Plasmodium*. Nat. Comm..

[24] Zhang HB, Howard EM, Roepe PD (2002). Analysis of the antimalarial drug resistance protein PfCRT expressed in yeast. J. Biol. Chem..

[25] Islahudin F, Pleass RJ, Avery SV, Ting K-N (2012). Quinine interactions with tryptophan and tyrosine in malaria patients, and implications for quinine responses in the clinical setting. J. Antimicr. Chemother..

[26] Thompson AJ, Lummis SCR (2008). Antimalarial drugs inhibit human 5-HT_3_ and GABA_A_ but not GABA_C_ receptors. Brit. J. Pharmacol..

[27] Modrzynska KK (2012). Quantitative genome re-sequencing defines multiple mutations conferring chloroquine resistance in rodent malaria. BMC Genom..

[28] Wang ZL (2016). Genome-wide association analysis identifies genetic loci associated with resistance to multiple antimalarials in *Plasmodium falciparum* from China-Myanmar border. Sci. Rep..

[29] Cobbold SA, Llinas M, Kirk K (2016). Sequestration and metabolism of host cell arginine by the intraerythrocytic malaria parasite *Plasmodium falciparum*. Cellul. Microbiol..

[30] Khozoie C, Pleass RJ, Avery SV (2009). The antimalarial drug quinine disrupts Tat2p-mediated tryptophan transport and causes tryptophan starvation. J Biol Chem.

[31] Lakowicz, J. R. *Principles of Fluorescence Spectroscopy*. 3 edn, (Springer-Verlag, 2006).

[32] Umebayashi K, Nakano A (2003). Ergosterol is required for targeting of tryptophan permease to the yeast plasma membrane. J. Cell Biol..

[33] Moura, P. A. *Novel insights into the digestive vacuole biology of the malarial parasite Plasmodium falciparum*. PhD thesis, Yeshiva University, (2008).

[34] Holland SL, Avery SV (2009). Actin-mediated endocytosis limits intracellular Cr accumulation and Cr toxicity during chromate stress. Toxicol. Sci..

[35] Guizetti J, Scherf A (2013). Silence, activate, poise and switch! Mechanisms of antigenic variation in. Plasmodium falciparum. Cellul. Microbiol..

[36] Hewitt SK, Foster DS, Dyer PS, Avery SV (2016). Phenotypic heterogeneity in fungi: Importance and methodology. Fung. Biol. Rev..

[37] Symmons O, Raj A (2016). What’s luck got to do with it: single cells, multiple fates, and biological nondeterminism. Mol. Cell.

[38] Halliwell SC, Smith MCA, Muston P, Holland SL, Avery SV (2012). Heterogeneous expression of the virulence-related adhesin Epa1 between individual cells and strains of the pathogen. Candida glabrata. Euk. Cell.

[39] Siltberg-Liberles J, Grahnen JA, Liberles DA (2011). The evolution of protein structures and structural ensembles under functional constraint. Genes.

[40] Soding J, Biegert A, Lupas AN (2005). The HHpred interactive server for protein homology detection and structure prediction. Nucl. Acids Res..

[41] Jiang HY (2008). Genome-wide compensatory changes accompany drug-selected mutations in the *Plasmodium falciparum crt* gene. PLoS One.

[42] Martin RE, Henry RI, Abbey JL, Clements JD, Kirk K (2005). The ‘permeome’ of the malaria parasite: an overview of the membrane transport proteins of *Plasmodium falciparum*. Genome Biol..

[43] Wilson DW, Langer C, Goodman CD, McFadden GI, Beeson JG (2013). Defining the timing of action of antimalarial drugs against *Plasmodium falciparum*. Antimicr. Ag. Chemother..

[44] Kolakovich KA, Gluzman IY, Duffin KL, Goldberg DE (1997). Generation of hemoglobin peptides in the acidic digestive vacuole of *Plasmodium falciparum* implicates peptide transport in amino acid production. Mol. Biochem. Parasitol..

[45] O’Connor SE, Maresh JJ (2006). Chemistry and biology of monoterpene indole alkaloid biosynthesis. Natural Product Rep..

[46] Avery SV (2006). Microbial cell individuality and the underlying sources of heterogeneity. Nat. Rev. Microbiol..

[47] Brock A, Chang H, Huang S (2009). Non-genetic heterogeneity - a mutation-independent driving force for the somatic evolution of tumours. Nature Rev. Genet..

[48] Wood TK (2016). Combatting bacterial persister cells. Biotechnol. Bioeng..

[49] Codd A, Teuscher F, Kyle DE, Cheng Q, Gatton ML (2011). Artemisinin-induced parasite dormancy: a plausible mechanism for treatment failure. Malaria J..

[50] Fidock DA (2000). Mutations in the *P*. *falciparum* digestive vacuole transmembrane protein PfCRT and evidence for their role in chloroquine resistance. Mol. Cell.

[51] Juge N (2015). *Plasmodium falciparum* chloroquine resistance transporter is a H^+^-coupled polyspecific nutrient and drug exporter. Proc. Natl. Acad. Sci. USA.

[52] Cooper RA (2007). Mutations in transmembrane domains 1, 4 and 9 of the *Plasmodium falciparum* chloroquine resistance transporter alter susceptibility to chloroquine, quinine and quinidine. Mol. Microbiol..

[53] Richards SN (2016). Molecular mechanisms for drug hypersensitivity induced by the malaria parasite’s chloroquine resistance transporter. PLoS Pathog..

[54] Golassa L, Erko B, Baliraine FN, Aseffa A, Swedberg G (2015). Polymorphisms in chloroquine resistance-associated genes in *Plasmodium vivax* in Ethiopia. Malaria J..

[55] Nomura T (2001). Evidence for different mechanisms of chloroquine resistance in 2 *Plasmodium* species that cause human malaria. J. Infect. Dis..

[56] Melo GC (2014). Expression levels of *pvcrt-o* and *pvmdr-1* are associated with chloroquine resistance and severe *Plasmodium vivax* malaria in patients of the Brazilian Amazon. PLoS One.

[57] Popovici J, Menard D (2015). Challenges in antimalarial drug treatment for vivax malaria control. Trends Mol. Med..

[58] Hunt P (2010). Experimental evolution, genetic analysis and genome re-sequencing reveal the mutation conferring artemisinin resistance in an isogenic lineage of malaria parasites. BMC Genom..

[59] Jones DT (1999). Protein secondary structure prediction based on position-specific scoring matrices. J. Mol. Biol..

[60] Moreno-Martinez E, Vallieres C, Holland SL, Avery SV (2015). Novel, synergistic antifungal combinations that target translation fidelity. Sci. Rep..

[61] Gari E, Piedrafita L, Aldea M, Herrero E (1997). A set of vectors with a tetracycline-regulatable promoter system for modulated gene expression in *Saccharomyces cerevisiae*. Yeast.

[62] Gietz RD, Woods RA (2002). Transformation of yeast by lithium acetate/single-stranded carrier DNA/polyethylene glycol method. Methods Enzymol..

[63] Longtine MS (1998). Additional modules for versatile and economical PCR-based gene deletion and modification in *Saccharomyces cerevisiae*. Yeast.

[64] Ausubel, F. M. *et al*. *Current Protocols in Molecular Biology*. (John Wiley & Sons, 2007).

[65] Halliwell SC, Smith MC, Muston P, Holland SL, Avery SV (2012). Heterogeneous expression of the virulence-related adhesin Epa1 between individual cells and strains of the pathogen *Candida glabrata*. Eukaryot. Cell.

[66] Vallieres C, Holland SL, Avery SV (2017). Mitochondrial ferredoxin determines vulnerability of cells to copper excess. Cell Chem. Biol..

[67] dos Santos SC, Tenreiro S, Palma M, Becker J, Sa-Correia I (2009). Transcriptomic profiling of the *Saccharomyces cerevisiae* response to quinine reveals a glucose limitation response attributable to drug-induced inhibition of glucose uptake. Antimicr. Ag. Chemother..

[68] Islahudin F (2013). Cell wall perturbation sensitizes fungi to the antimalarial drug chloroquine. Antimicr. Ag. Chemother..

