# Supplementary material for: Heterologous Expression of a Novel Drug Transporter from the Malaria Parasite Alters Resistance to Quinoline Antimalarials
Source: Sci Rep. 2018 Feb 6;8:2464. doi: 10.1038/s41598-018-20816-0 (PMC5802821; doi:10.1038/s41598-018-20816-0)
Supplement: Supplementary file 1 — Supplementary material [file 41598_2018_20816_MOESM1_ESM.pdf]

SUPPLEMENTARY INFORMATION:

Heterologous Expression of a Novel Drug Transporter from the Malaria Parasite Alters Resistance to Quinoline Antimalarials

Sarah M. Tindall[†,1], Cindy Vallières[†,1], Dev H. Lakhani[1],

Farida Islahudin[2], Kang-Nee Ting[3], Simon V. Avery*[,1]

[†]These authors contributed equally to this work

1. School of Life Sciences, University of Nottingham, University Park, Nottingham NG7 2RD, UK

2. Faculty of Pharmacy, Universiti Kebangsaan, Malaysia

3. Department of Biomedical Sciences, University of Nottingham Malaysia Campus, Semenyih, Malaysia

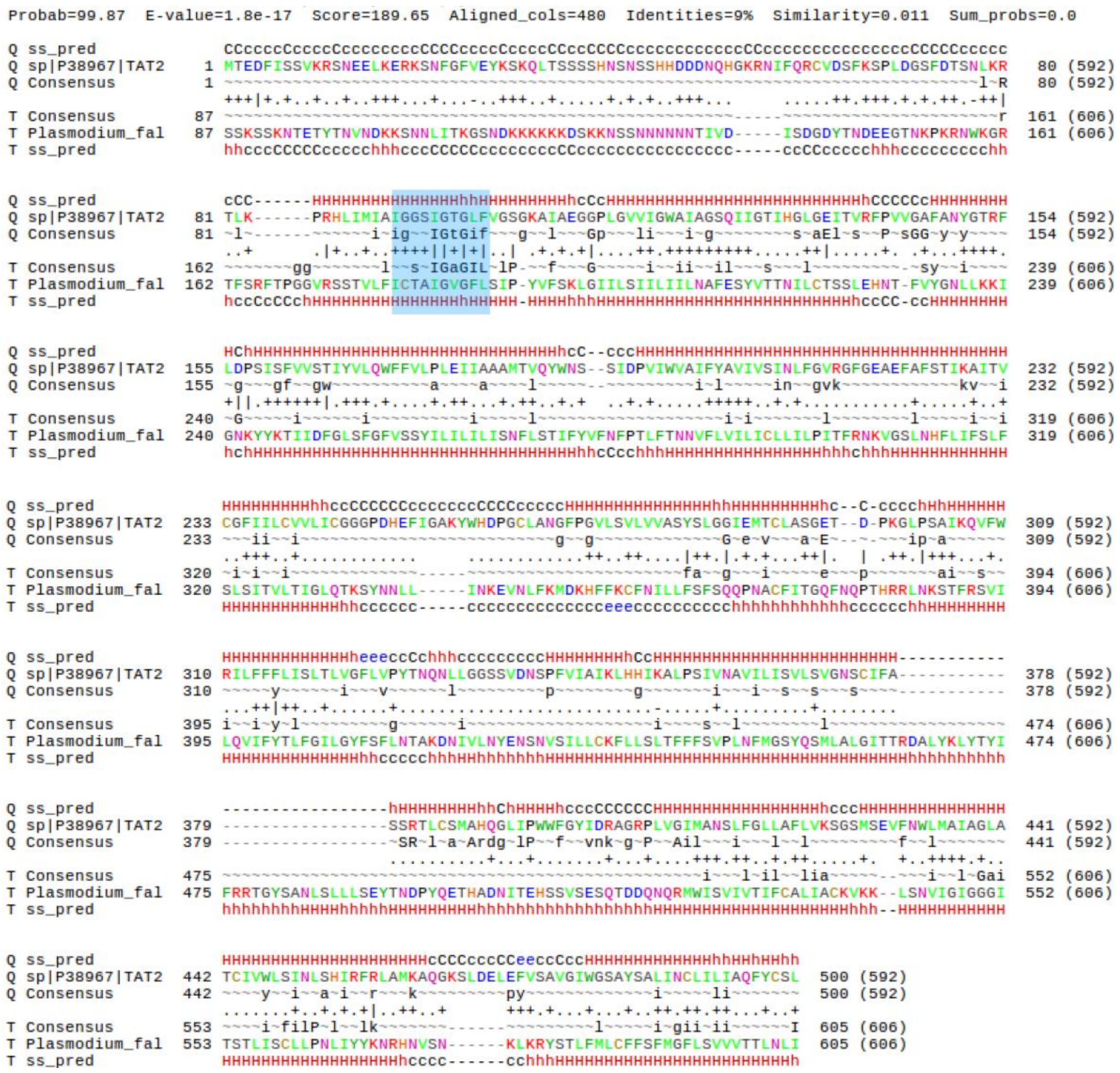

FIG S1 HHPRED homology detection and structure prediction. Profile alignment *S. cerevisiae*Tat2 and *P. falciparum* PF3D7_0629500 (PlasmoDB: PFF1430c , Uniprot ID:C6KTD0). The symbols + and | signify good and very good conservation matches respectively. For more information about interpreting results, see http://toolkit.tuebingen.mpg.de/hhpred/help_results. (The manually annotated region in blue below indicates a section of very good structural and possibly functional conservation).

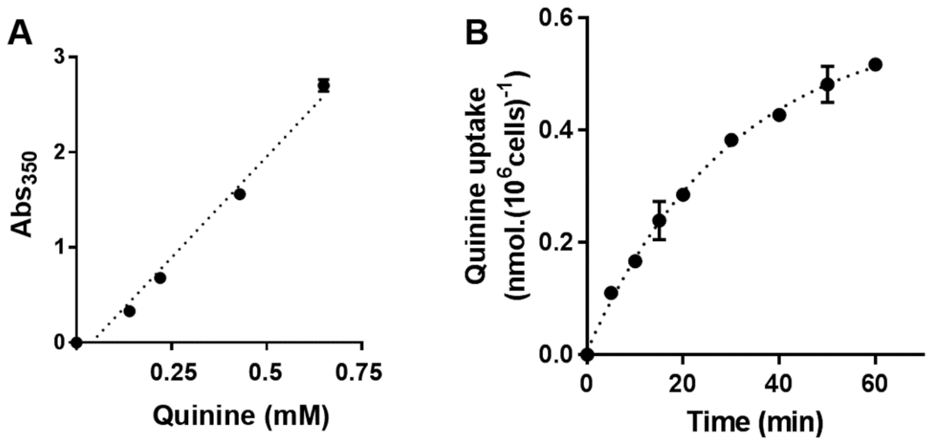

FIG S2. Linearity of quinine determinations and saturation of uptake. (**A**) Quinine solutions of various concentrations were assayed by absorbance measurements at 350 nm. (**B**) Wild type yeast was incubated for 60 min with 4 mM quinine. Quinine analysis in cell lysates was according to absorbance determinations at 350 nm, normalised for cell numbers determined just before lysis, with subtraction of background (minus-quinine). Values are means ±SEM from three independent determinations.

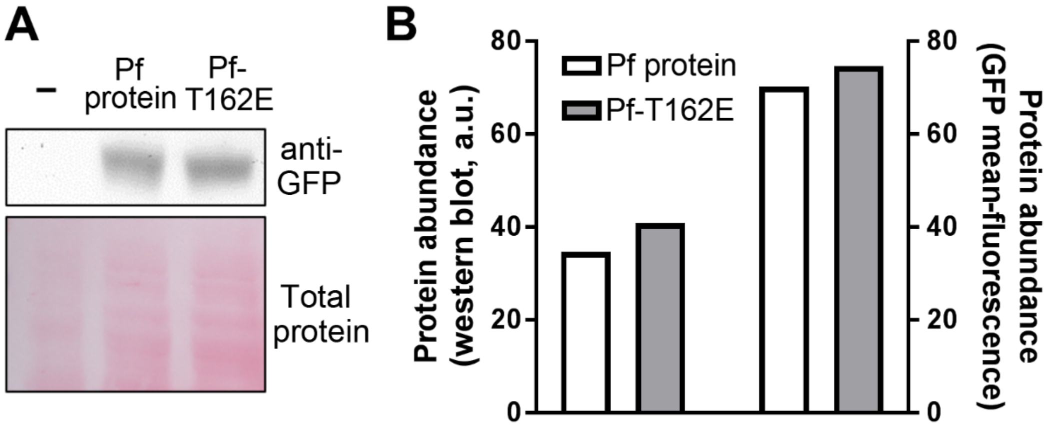

FIG S3. Similar expression levels of the PF3D7-0629500 and PF3D7-0629500^{T162E} proteins. (**A**) Protein was extracted from yeast *tat2Δ* cells transformed with pCM190 vector, either empty (–) or expressing GFP tagged versions of PF3D7-0629500 (Pf protein) or the same protein carrying the T162E SNP (Pf-T162E), after culturing in the absence of doxycycline to maximize expression. (The *tat2Δ* background was used here to eliminate any potential effect of expression of the yeast homologue). The GFP-tagged parasite protein was detected by western blotting with anti-GFP antibody, and total protein loading indicated by Ponceau S staining. a.u., arbitrary units. (**B**) Left bars, quantification of anti-GFP bands (A) by densitometry, with normalization against the level of total-protein staining; Right bars, quantification of GFP fluorescence in ~10,000 cells expressing the GFP-tagged wild type or SNP versions of the parasite protein by flow cytometry.
